# 

**Published:** 1898-09

**Authors:** 


LEADS VETERINARY JOURNALISM IN AMERICA.

Vol. XIX. SEPTEMBER, 1898. No. 9.

PUBLISHED MONTHLY AT $3 A YEAR. SINGLE COPIES, 30 CENTS.
FOREIGN SUBSCRIPTIONS $3.50 PER YEAR.

### THE JOURNAL OF COMPARATIVE MEDICINE AND VETERINARY ARCHIVES

EDITED AND PUBLISHED BY
RUSH SHIPPEN HUIDEKOPER, Veterinarian (Alfort),
W. HORACE HOSKINS, D.V.S.,
H. D. GILL, V.S.

ALL COMMUNICATIONS AND PAYMENTS SHOULD BE ADDRESSED TO
**Dr. W. Horace Hoskins, Managing Editor, 3452 Ludlow St., Philadelphia, Pa.**
Copies may be had on order of all news-dealers at home and abroad.

AN ADVERTISING MEDIUM UNEQUALLED IN THE VETERINARY FIELD.

